# The Role of Nonshivering Thermogenesis Genes on Leptin Levels Regulation in Residents of the Coldest Region of Siberia

**DOI:** 10.3390/ijms22094657

**Published:** 2021-04-28

**Authors:** Alena A. Nikanorova, Nikolay A. Barashkov, Vera G. Pshennikova, Sergey S. Nakhodkin, Nyurgun N. Gotovtsev, Georgii P. Romanov, Aisen V. Solovyev, Sargylana S. Kuzmina, Nikolay N. Sazonov, Sardana A. Fedorova

**Affiliations:** 1 Laboratory of Molecular Genetics, Yakut Science Centre of Complex Medical Problems, 677010 Yakutsk, Sakha Republic (Yakutia), Russia; nikanorova.alena@mail.ru (A.A.N.); psennikovavera@mail.ru (V.G.P.); donzcrew@mail.ru (N.N.G.); gpromanov@gmail.com (G.P.R.); nelloann@mail.ru (A.V.S.); 2 Laboratory of Molecular Biology, M.K. Ammosov North-Eastern Federal University, 677000 Yakutsk, Sakha Republic (Yakutia), Russia; sergnahod@mail.ru (S.S.N.); sskuzmina@bk.ru (S.S.K.); saznikol@mail.ru (N.N.S.); sardaanafedorova@mail.ru (S.A.F.)

**Keywords:** leptin, nonshivering thermogenesis, UCP1, cold climate, adaptation, adipose tissue, Yakut population, Siberia, Russia

## Abstract

Leptin plays an important role in thermoregulation and is possibly associated with the microevolutionary processes of human adaptation to a cold climate. In this study, based on the Yakut population (*n* = 281 individuals) living in the coldest region of Siberia (t°minimum −71.2 °C), we analyze the serum leptin levels and data of 14 single nucleotide polymorphisms (SNPs) of 10 genes (*UCP1*, *UCP2*, *UCP3*, *FNDC5*, *PPARGC1A*, *CIDEA*, *PTGS2*, *TRPV1*, *LEPR*, *BDNF*) that are possibly involved in nonshivering thermogenesis processes. Our results demonstrate that from 14 studied SNPs of 10 genes, 2 SNPs (the TT rs3811787 genotype of the *UCP1* gene and the GG rs6265 genotype of the *BDNF* gene) were associated with the elevated leptin levels in Yakut females (*p* < 0.05). Furthermore, of these two SNPs, the rs3811787 of the *UCP1* gene demonstrated more indications of natural selection for cold climate adaptation. The prevalence gradient of the T-allele (rs3811787) of *UCP1* increased from the south to the north across Eurasia, along the shore of the Arctic Ocean. Thereby, our study suggests the potential involvement of the *UCP1* gene in the leptin-mediated thermoregulation mechanism, while the distribution of its allelic variants is probably related to human adaptation to a cold climate.

## 1. Introduction

Leptin is a 16 kDa peptide hormone composed of 167 amino acids and is a member of the type I helical cytokine family [1,2]. In humans, leptin is encoded by the *LEP* gene located on chromosome seven [3]. Leptin is produced primarily by the adipocytes in adipose tissue of humans and other mammals [4,5]. The major function of leptin is the modulation of food intake [6,7,8], along with energy homeostasis [9,10]. However, several studies have shown that leptin also plays an important role in thermoregulation [11,12,13,14]. It was found that leptin-deficient *ob*/*ob* mice display hypothermia, and as a result, such mice are unable to withstand prolonged cold exposure [11,12,13]. It was later determined that exogenous administration of leptin optimizes the body temperature of *ob*/*ob* mice [15,16,17,18], indicating an involvement of leptin on thermoregulation. Exogenous administration of leptin to *ob*/*ob* mice increases their body temperature without changing energy expenditure [19]. In humans with a hereditary leptin deficiency caused by mutations in the *LEP* gene, leptin treatment also did not affect energy expenditure [20,21]. It is possible that leptin affects heat exchange [19] and/or shifts in thermoregulatory thresholds [19,22].

Thermoregulation is one of the most important mechanisms in the physiology of mammals. It is crucial for preventing cellular damage from physiologically extreme temperatures and also for optimizing biological activity and, consequently, body function [14]. In response to exposure to cold, nonshivering thermogenesis increases metabolic heat production above the normal basal level [23]. It takes place mainly in brown adipose tissue (BAT) and to a lesser degree in skeletal muscle, liver, brain, and white adipose tissue (WAT) [24,25]. BAT protects mammals from hypothermia and counteracts metabolic diseases [26]. However, the BAT is facultative and is activated only under prolonged cold exposure [27,28]. In 2015 in the Sakha Republic (eastern Siberia, Russia), BAT was found in an adult individual who was continuously exposed to extremely low temperatures [29]. The BAT was detected in adipose tissue samples from paraaortic, perirenal, subclavian, and parathyroid areas [29]. These findings indicate that people living in cold climatic conditions have actively functioning BAT in adulthood as an evolutionary adaptation to inhabiting Arctic regions of the world. The Sakha Republic is considered to be the coldest region of Siberia, with 40% of its area located above the Arctic Circle in the permafrost zone. The minimum air temperature in this part of Siberia is around −71.2 °C. Most of the residents (466,492 people according to the Russian Census, 2010) of the Sakha Republic are the indigenous Turkic-speaking population of Yakuts (originally named the Sakha). Previous studies have shown that the indigenous peoples of Siberia have developed certain metabolic and physiological features in order to adapt to the extreme climatic conditions, such as lower lipid levels in blood serum, higher levels of energy metabolism [30], higher blood pressure levels [24,31,32,33], and seasonal variation in free thyroid hormones in the blood, which declines during the winter [34]. Furthermore, the recent meta-analysis study revealed that males living in northern regions have higher blood leptin levels compared to males living in southern regions of the world [35].

Thus, we hypothesize that the indigenous peoples of Siberia may have developed genetic features regulating blood leptin levels as an adaptation to the cold climate. Therefore, the aim of this study is to identify the relationship between nonshivering thermogenesis-related genes and the leptin blood levels in the Yakut population living in the coldest region of Siberia.

## 2. Results

### 2.1. Serum Leptin Levels in the Yakut Population

**Figure 1:**
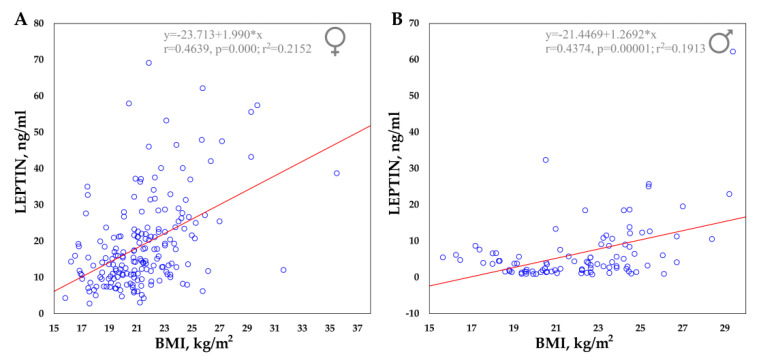
Correlation between serum leptin concentrations and BMI in the Yakut population. (**A**) Females. (**B**) Males.

All the data described in the study are provided in the Supplementary Table S1. The mean serum leptin concentration was 18.87 ± 12.31 ng/mL in females and 6.56 ± 8.47 ng/mL in males. Gender differences in leptin levels were observed in the Yakut population, with females having higher leptin levels than males (*p* < 0.01). A statistically significant linear correlation between serum leptin levels and body mass index (BMI) in females (r^2^ = 0.2152; *p* < 0.01) and males (r^2^ = 0.1913; *p* < 0.01) was found, where leptin levels would increase with increasing BMI (Figure 1).

Table 1 presents characteristics of the sample (*n* = 281), divided into three groups by BMI and gender. Males displayed significantly higher weight and height than females (*p* = 0.01) in all three groups. Males with a normal BMI displayed a significantly higher BMI than females (*p* = 0.03). Gender differences in leptin levels were detected in all three groups (*p* < 0.01).

### 2.2. Association between Leptin Levels and 14 SNP Markers of the 10 Genes, Potentially Related to Nonshivering Thermogenesis Processes

The genotypes and the allele frequencies of the 14 SNP markers of the 10 genes potentially related to nonshivering thermogenesis processes (*UCP1*, *UCP2*, *UCP3*, *FNDC5*, *LEPR*, *PTGS2*, *TRPV1*, *BDNF*, *CIDEA*, *PPARGC1A)* are presented in Supplementary Table S2. Serum leptin levels were different between genders (*p* < 0.01) and in direct correlation with BMIs (*p* < 0.01). Therefore, to correctly assess the association between the leptin level and the studied genotypes, we made stratification given the gender differences and the weight-related differences. The complete data of the one-factor analysis of variance (ANOVA) are presented in Supplementary Table S3.

**Figure 2:**
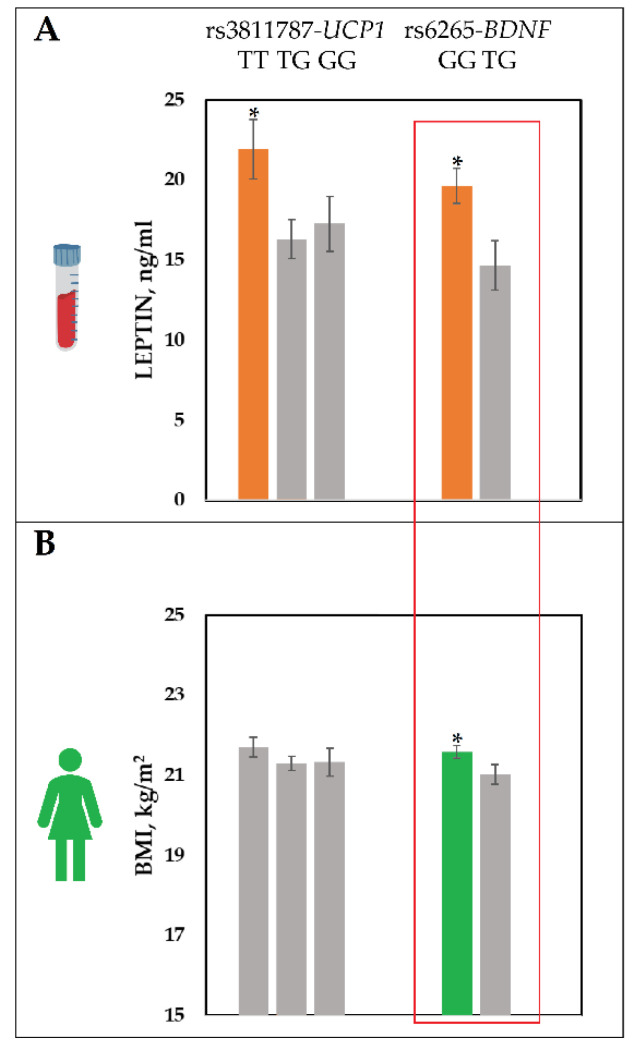
The leptin levels and BMI divided by the rs3811787 (*UCP1*) and the rs6265 (*BDNF*) genotypes. (**A**) Comparison of leptin levels by the rs3811787 (*UCP1*) and rs6265 (*BDNF*) genotypes for the group of females with normal BMI (*n* = 215). (**B**) Comparison of BMI by the rs3811787 (*UCP1*) and rs6265 (*BDNF*) genotypes for the group of females with normal BMI (*n* = 215). The data shown are mean ± SE. * *p* ≤ 0.05.

This analysis revealed no association between leptin levels and the studied genotypes in males (Table S3). In females, associations between leptin levels and studied genotypes were found for the two SNPs: rs3811787 (*UCP1*) (*p* = 0.02) and rs6265 (*BDNF*) (*p* = 0.01). For the rs3811787 (*UCP1)*, leptin levels were higher in the TT homozygotes (21.94 ± 12.87 ng/mL) compared to the GT heterozygotes (16.31 ± 10.33 ng/mL) and the GG homozygotes (17.27 ± 9.19 ng/mL) (*p* = 0.02) (Figure 2A). For the rs6265 of the *BDNF* gene, leptin levels were higher in females with the GG genotype (19.64 ± 11.05 ng/mL) compared to the GA heterozygotes (14.65 ± 10.01 ng/mL) (*p* = 0.01) (Figure 2A). 

An additional analysis was performed in order to identify the relationship of BMI, weight, and height with the genotypes of the two studied SNPs (rs3811787—*UCP1*, rs6265—*BDNF*) in females with a normal BMI (Supplementary Table S4). Associations were found between rs6265 (*BDNF*) and the BMI of females (*p* = 0.05) (Figure 2B). For rs3811787 of the *UCP1* gene, no significant associations with BMI, weight, or height were found (Figure 2B).

### 2.3. Search for Indicators of Natural Selection for Cold Climate Adaptation 

The polymorphisms that were identified to be associated with leptin levels in the Yakut population (rs3811787—*UCP1*, rs6265—*BDNF*) were studied for possible indicators of natural selection towards cold climate adaptation. We used the “1000 Genomes Project” [36] database for a comparative analysis of the prevalence of the polymorphisms in eight East Asian populations living in different climatic zones (Supplementary Table S5 and S6). The sample of Yakuts (*n* = 281) consisted of three subgroups: northern (N.YAK, *n* = 16) from the subarctic climate zone, Vilyuy (V.YAK, *n* = 67), and central (C.YAK, *n* = 198) from temperate climate zones, and all of them were combined into the “North Asia” group. Other East Asian populations from temperate (Han Chinese—CHB), subtropical (Japanese—JPT; southern Han Chinese—CHS; Chinese Dai—CDX), and subequatorial (Vietnamese—KHV) climate zones were combined into the “South Asia” group. The prevalence of the G-allele of rs6265 (*BDNF*) was found to be significantly higher in the “North Asia” group (83%, CI: 0.779–0.862) compared to the “South Asia” group (51%, CI: 0.471–0.552) (*p* = 0.01). Furthermore, the prevalence of the T-allele of rs3811787 (*UCP1*) was significantly higher in the “North Asia” group (63%, CI: 0.546–0.652) compared to the “South Asia” group (49%, CI: 0.448–0.529) (*p* = 0.02).

**Figure 3:**
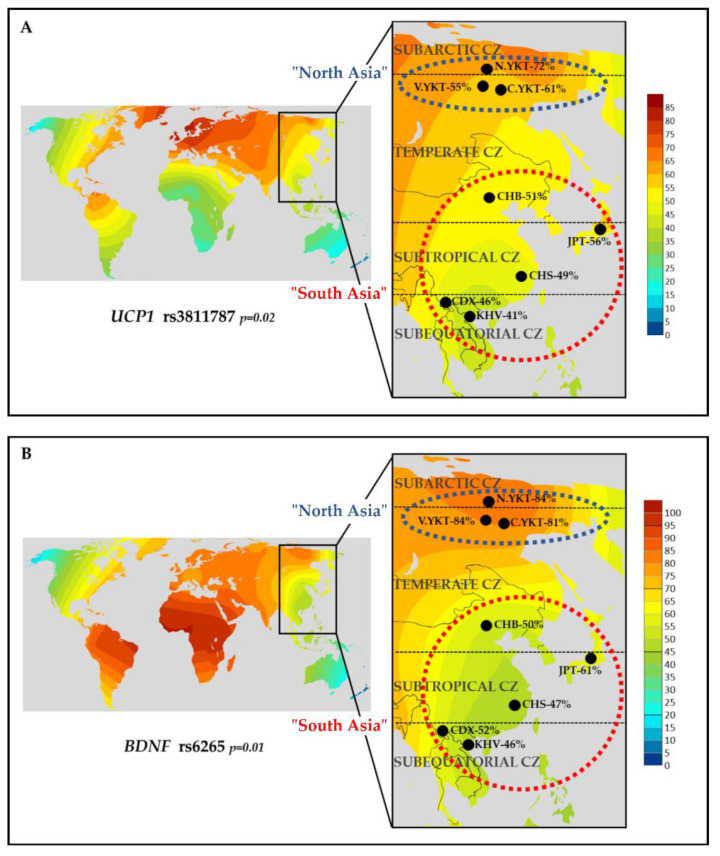
The search for indicators of natural selection for cold climate adaptation. (**A**) T-allele, rs3811787 (*UCP1*). (**B**) G-allele, rs6265 (*BDNF*). The allele prevalence gradients are indicated on the color scale. CZ—climatic zone; “North Asia”: N.YAK—northern Yakuts, V.YAK—Vilyuy Yakuts, C.YAK—central Yakuts; “South Asia”: CHB—Han Chinese, JPT—Japanese, CHS—southern Han Chinese, CDX—Chinese Dai, KHV—Vietnamese.

We constructed a map with the worldwide distribution of allele prevalence (rs3811787 T-allele, rs6265 G-allele). The highest prevalence of the T-allele of rs3811787 (*UCP1* gene) was observed in the northern regions of Europe (Figure 3A). We found that the T-allele of rs3811787 (*UCP1* gene) gradient increases from the south to the north in Eurasia along the shore of the Arctic Ocean. The highest prevalence of the G-allele of rs6265 (*BDNF* gene) was registered in the equatorial part of Africa. The gradient decreases towards the north and the east from Africa (Figure 3B). However, we can see the peak of a gradient of the G-allele in Siberia (Figure 3B).

## 3. Discussion

In this study, based on the Yakut population living in the coldest region of Siberia (t°minimum −71.2 °C), we analyze serum leptin levels and data on 14 single nucleotide polymorphisms (SNPs) of 10 genes (*UCP1*, *UCP2*, *UCP3*, *FNDC5*, *PPARGC1A*, *CIDEA*, *PTGS2*, *TRPV1*, *LEPR*, *BDNF*) that are possibly involved in nonshivering thermogenesis processes. These genes were selected based on the available literature data on nonshivering thermogenesis activation mechanisms and genome-wide analysis of cold adaptation in humans [37,38,39,40,41,42,43,44,45]. The leptin levels were three times higher in females (18.87 ± 12.31 ng/mL) than in males (6.56 ± 8.47 ng/mL) (*p* < 0.01). We also demonstrated the strong direct correlation of increased leptin levels with a high BMI in females (r^2^ = 0.2152; *p* < 0.01) and in males (r^2^ = 0.1913; *p* < 0.01) (Figure 1). Our results are in accordance with the previously published data on the direct positive correlation of leptin blood levels with BMI and, and on gender differences [4,46,47,48,49,50,51,52,53,54,55,56,57,58,59,60].

Considering the obtained results, in order to correctly identify the association between leptin levels and the genotypes of 14 SNPs of the 10 genes potentially associated with thermoregulation processes (*UCP1*, *UCP2*, *UCP3*, *FNDC5*, *LEPR*, *PTGS2*, *TRPV1*, *BDNF*, *CIDEA*, *PPARGC1A*), the sample was stratified by BMI and gender. In females, out of 14 studied SNP markers, 2 polymorphisms (rs3811787, *UCP1* gene and rs6265, *BDNF* gene) had a statistically significant association with the leptin levels (*p* < 0.05) (Supplementary Table S3). Regarding rs3811787 (*UCP1)*, leptin levels were higher in the TT homozygotes (21.94 ± 12.87 ng/mL) compared to the GT heterozygotes (16.31 ± 10.33 ng/mL) and the GG homozygotes (17.27 ± 9.19 ng/mL) (*p* = 0.02). Regarding rs6265 (*BDNF*), leptin levels were higher in females with the GG genotype (19.64 ± 11.05 ng/mL) compared to the GA heterozygotes (14.65 ± 10.01 ng/mL) (*p* = 0.01). Additionally, females with the GG genotype had significantly higher BMIs (21.59 ± 1.6 kg/m^2^) than those with the GA genotype (21.02 ± 1.6 kg/m^2^) (*p* = 0.05) (Supplementary Table S4). In males, there was no correlation between leptin levels and the studied SNP markers.

A subsequent search for signals of adaptation to the cold climate was performed for two SNPs (rs3811787 of the *UCP1* gene and rs6265 of the *BDNF* gene) that were found to be associated with the leptin levels. Using the open database “1000 Genomes Project” [36], allele frequencies were analyzed between populations living in relatively cold climates (the “North Asia” group—subarctic and temperate climatic zone) and populations living in relatively warm climates (the “South Asia” group—subtropical and subequatorial climatic zone) (Supplementary Table S5 and S6). The search for possible indicators of adaptation to cold climates showed that the prevalence of the G-allele of rs6265 (*BDNF*) in the “North Asia” group (83%, CI: 0.779–0.862) was significantly higher than in the “South Asia” group (51%, CI: 0.471–0.552) (*p* = 0.01). Furthermore, the frequency of the T-allele of rs3811787 (*UCP1*) in the “North Asia” group (63%, CI: 0.546–0.652) was significantly higher compared to the “South Asia” group (49%, CI: 0.448–0.529) (*p* = 0.02). The search for indicators of natural selection for cold climate adaptation shows that the T-allele of rs3811787 of the *UCP1* gene has the strongest association with an adaptation to cold, as the frequency of this allele increases in northern Europe and Siberia and is associated with northern regions of Eurasia. The global distribution of the rs6265 polymorphism of the *BDNF* gene suggests that identified indicators of natural selection for the Yakut population may be attributed to the functional importance of the gene product, which is associated with other adaptation mechanisms that are less strongly related to the adaptation to cold.

In order to describe a potential UCP1-leptin interaction pathway, we studied leptin-dependent neuro-fatty regulation of nonshivering thermogenesis. The *UCP1* (uncoupling protein 1) gene encodes thermogenin, an adipocyte-specific mitochondrial protein. The UCP1 uncouples respiration from ATP synthesis and therefore provokes energy dissipation in the form of heat, while also stimulating high levels of fatty acid oxidation [26,61]. It is known that UCP1 is associated with leptin [62], however, the control UCP1 has over the mechanism of leptin-dependent neuro-adipose communication remains insufficiently understood. Leptin signaling regulates the plasticity of sympathetic adipose tissue structure via a descending nerve pathway, which is crucial for energy homeostasis [63]. It is known that leptin’s effects are mediated through the orexigenic neuropeptide Y (NPY), agouti-related peptide (AgRP) neurons, and also through proopiomelanocortin (POMC) neurons in the arcuate nucleus of the hypothalamus [46,64,65,66,67]. These AgRP- and POMC-related neurons act via neurons (BDNF^PVH^) that express BDNF in the paraventricular nucleus of the hypothalamus [63]. Leptin and its receptors in the arcuate nucleus of the hypothalamus increase the activity of AgRP and POMC neurons via BDNF^PVH^, triggering the production and release of the α-melanocyte-stimulating hormone. This hormone activates melanocortin-3 and melanocortin-4 receptors, which increases the sympathetic nervous system’s activity [10]. These effects of leptin and its receptors in the hypothalamus increase both the UCP1 expression and the brown adipose tissue activity [62,68]. 

**Figure 4:**
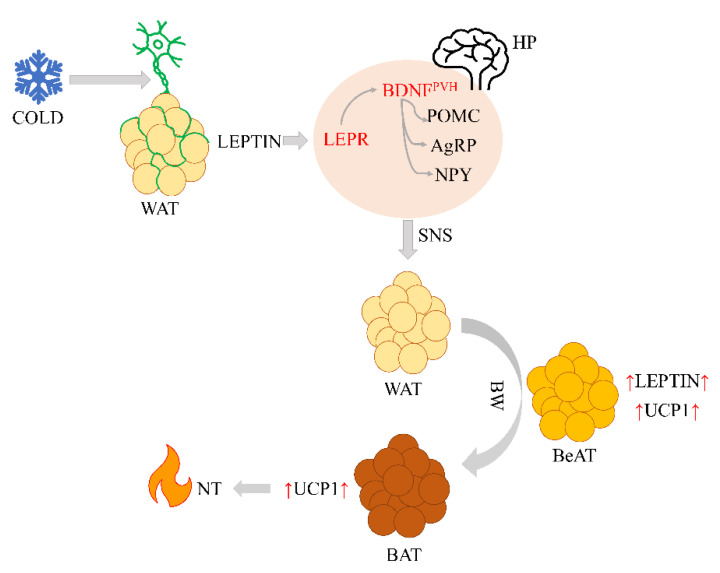
Possible mechanism of leptin-dependent neuro-fatty regulation of nonshivering thermogenesis. Note: WAT—white adipose tissue, LEPR—leptin receptor, BDNF^PVH^—BDNF neuron, AgRP—agouti-related peptide neuron, NPY—neuropeptide Y, POMC—proopiomelanocortin, HP—the hypothalamus, SNS—sympathetic nervous system, BW—browning, BeAT—beige adipose tissue, BAT—brown adipose tissue, NT—nonshivering thermogenesis, red up-arrows—increased levels of leptin or UCP1.

We hypothesize that white adipocytes undergo browning in response to prolonged cold exposure, which increases the levels of the UCP1 protein (Figure 4). 

A study by Commins et al. [62] showed that administration of exogenous leptin to *ob*/*ob* mice resulted in a 4–5-fold increase in mRNA levels of the UCP1 protein in BAT. Efremova et al. [69] found that a constant part of the mediastinal and perirenal fat (up to about 40%) in adult residents of eastern Siberia had the morphology typical of brown adipocytes and that a relevant percentage (up to about 30%) expressed the functional marker of UCP1. This study indicates the possible activation of nonshivering thermogenesis in response to extremely low temperatures in Siberia as part of the evolutionary adaptation mechanism of humans to cold climates.

## 4. Materials and Methods

### 4.1. Subjects

The research sample comprised 281 people: 186 females and 95 males (with a mean age of 19.8 ± 0.75 years), all of Yakut ethnicity. They presented no health issues at the time of the study and had completed a questionnaire in which they specified their gender, ethnicity, and age. All participants gave written informed consent for participation in the study. This study was approved by the local Biomedical Ethics Committee at the Yakut Scientific Center of Complex Medical Problems, Siberian Branch of the Russian Academy of Medical Sciences, Yakutsk, Russia (Yakutsk, Protocol No. 16, 13 December 2014).

### 4.2. Anthropometric Measurements

Venous blood was taken from all participants in the morning after an 8 h fast. Anthropometric parameters (body weight in kilograms, height in centimeters) were measured for all participants by standardized methods. BMI was calculated by dividing body mass by the square of the body height. The sample was divided into three groups by BMI, according to World Health Organization (WHO) guidelines [70]: underweight (≤18.49 kg/m^2^), normal weight (18.5–24.99 kg/m^2^), and overweight (≥25 kg/m^2^).

### 4.3. Serum Leptin Analyses

Fasting serum leptin levels (ng/mL) were determined with the human leptin sandwich enzyme-linked immunoassay (ELISA) “LEPTIN ELISA KIT” (Diagnostics Biochem Canada Inc., London, ON, Canada). The concentration of leptin in the samples was measured at the wavelength of 450 nm on a VICTOR X5 Multilabel Plate Reader (Perkin Elmer Inc., Waltham, MA, USA).

### 4.4. DNA Analysis

Genomic DNA was isolated from the blood using phenol–chloroform extraction. A total of 14 polymorphisms of 10 genes were genotyped using the polymerase chain reaction–restriction fragment length polymorphism (PCR-RFLP) method. PCR was performed on a BioRad T100 Thermal Cycler (Bio-Rad Laboratories, Inc., Hercules, CA, USA). The data, including the primer sequence, annealing temperature, PCR product size, and restriction enzymes, are presented in Supplementary Table S7.

### 4.5. Stratification of the Sample of Yakuts

Stratification by BMI and gender was performed to determine the relationship between leptin levels and the genotypes of 14 SNPs of the 10 genes (*UCP1*, *UCP2*, *UCP3*, *FNDC5*, *LEPR*, *PTGS2*, *TRPV1*, *BDNF*, *CIDEA*, *PPARGC1A*). The Yakut sample (*n* = 281) was divided into three BMI groups: underweight (*n* = 37), normal weight (*n* = 215), and overweight/obese (*n* = 29). Underweight and overweight/obese males and females were excluded from the analysis. A comparative analysis of blood leptin levels and genotypes of 14 SNPs of the 10 genes (*UCP1*, *UCP2*, *UCP3*, *FNDC5*, *LEPR*, *PTGS2*, *TRPV1*, *BDNF*, *CIDEA*, *PPARGC1A*) was performed separately for females (*n* = 144) and males (*n* = 71) with a normal BMI (*n* = 215). 

### 4.6. The Search for Indicators of Natural Selection

Data from the open database “1000 Genomes Project” [36] were used for the search of possible indicators of natural selection for cold climate adaptation. Data were extracted for the following 20 populations: Esan (Nigeria), Gambians (Gambia), Luhya (Webuye, Kenya), Mende (Sierra Leone), Yoruba (Ibadan, Nigeria), Finns (Finland), Britons (England and Scotland), Iberians (Spain), Tuscans (Italy), Bengalis (Bangladesh), Punjabis (Lahore, Pakistan), Sri Lankan Tamils (United Kingdom), Chinese Dai (Xishuangbanna, China), Han Chinese (Beijing, China), southern Han Chinese (China), Japanese (Tokyo, Japan), Vietnamese (Ho Chi Minh City, Vietnam), Puerto Ricans (Puerto Rico), Colombians (Medellin, Colombia), and Peruvians (Lima, Peru). Thus, the total sample size comprised 1979 individuals. Using Surfer 12.0 software (Golden Software, Golden, CO, USA), a map of the allele frequency distribution in populations of North and South America, Eurasia, and Africa was composed, which included data on the allele frequencies of these 20 populations.

### 4.7. Statistical Analysis

The obtained data were analyzed using Statistica 13.5, a statistical software program (TIBCO Software Inc., Palo Alto, CA, USA). Values of *p* ≤ 0.05 were considered statistically significant. Quantitative results are reported as the mean ±standard deviation. The association of BMI with leptin levels was assessed with the multiple regression analysis. Comparative analysis of the three BMI groups between males and females was performed with the Mann–Whitney U test for the underweight and overweight/obese groups (*n* < 30) and with the Student’s *t*-test for the individuals with normal weight (*n* > 60). To identify statistically significant associations between the genotypes of the 14 SNPs of the 10 gene variants and serum leptin concentrations, a one-factor analysis of variance (ANOVA) was performed. Correspondence between the frequencies of the 14 SNPs of the 10 gene variants and the Hardy–Weinberg equilibrium was determined using standard formulas.

## 5. Conclusions

(1) Our analyses showed the strong positive correlation of body mass index (BMI) and serum leptin levels in the Yakut population, both in females (r^2^ = 0.2152; *p* < 0.01) and in males (r^2^ = 0.1913; *p* < 0.01). Serum leptin was significantly higher in females (18.87 ± 12.31 ng/mL) than in males (6.56 ± 8.47 ng/mL). We found that the TT genotype of rs3811787 (*UCP1*) and the GG genotype of rs6265 (*BDNF*) were associated with the elevated leptin levels in females with a normal BMI (*p* < 0.05).
(2) The rs3811787 (*UCP1)* and rs6265 (*BDNF)* were studied for possible indicators of natural selection towards cold climate adaptation. Among eight East Asian populations, the high prevalence of the T-allele of rs3811787 (*UCP1*) and the G-allele of rs6265 (*BDNF*) was found in populations living in subarctic and temperate climatic zones, in comparison with populations from subtropical and subequatorial climate (*p* < 0.05).
(3) Subsequent analysis of worldwide data showed that the T-allele of rs3811787 (*UCP1*) gradient increases from the south to the north in Eurasia, along the shore of the Arctic Ocean, while the G-allele of rs6265 (*BDNF*) less strongly correlates with cold climates and is probably more related to other adaptation mechanisms. These results demonstrate the potential involvement of the *UCP1* gene in the leptin-mediated thermoregulation mechanism, while the distribution of its allelic variants is probably related to human adaptation to the cold climate.

## Figures and Tables

**Table 1:** Characteristics of study subjects by BMI and gender.

Characteristics	Underweight (*n* = 37)		*p* ^1^	Normal Weight (*n* = 215)		*p* ^2^	Overweight/Obese (*n* = 29)		*p* ^1^
	F (*n* = 26)	M (*n* = 11)		F (*n* = 144)	M (*n* = 71)		F (*n* = 16)	M (*n* = 13)	
Age (years)	19.12 ± 1.45	18.91 ± 0.83	0.84	19.81 ± 2.1	20.1 ± 2.09	0.33	20.56 ± 2.37	20.38 ± 1.61	0.97
Weight (kg)	44.88 ± 3.64	50.45 ± 3.42	**0.01**	55.53 ± 5.8	66.11 ± 7.41	**0.01**	72.75 ± 11.13	81.46 ± 8.3	**0.01**
Height (cm)	160.23 ± 5.04	170.36 ± 5.89	**0.01**	160.92 ± 6.03	173.39 ± 5.96	**0.01**	162.19 ± 4.96	174.69 ± 6.64	**0.01**
BMI (kg/m^2^)	17.46 ± 0.72	17.39 ± 0.91	0.87	21.42 ± 1.62	21.96 ± 1.9	**0.03**	27.56 ± 2.88	26.64 ± 1.49	0.54
Leptin (ng/mL)	13.35 ± 8.2	5.64 ± 1.58	**0.0006**	18.18 ± 10.96	4.87 ± 5.5	**0.0001**	34 ± 17.59	16.62 ± 16.01	**0.009**

^1^ Mann–Whitney U criterion; ^2^ Student’s *t*-test; F—females; M—males.

## Data Availability

The data presented in this study are available on request from the corresponding author.

## Supplementary Materials

The following are available online at https://www.mdpi.com/article/10.3390/ijms22094657/s1.
