# Supplementary material for: The Role of Nonshivering Thermogenesis Genes on Leptin Levels Regulation in Residents of the Coldest Region of Siberia
Source: Int J Mol Sci. 2021 Apr 28;22(9):4657. doi: 10.3390/ijms22094657 (PMC8124869; doi:10.3390/ijms22094657)
Supplement: Supplementary file 1 [file ijms-22-04657-s001.zip › ijms-1185588-supplementary.pdf]

Supplementary Material

Table S1. Anthropometric characteristics and results of genotyping and ELISA analysis in 281 individuals of Yakuts

No	#DNA	Gender	Height, cm	Weight, kg	BMI, kg/m^2	Age, years	Leptin, ng/ml	*UCP1* rs1800592	*UCP1* rs3811787	*UCP2* rs659366	*UCP2* rs660339	*UCP3* rs1800849	*UCP3* rs2075577	*FNDC5* rs16835198	*FNDC5* rs3480	*LEPR* rs1137101	*PTGS2* rs689466	*TRPV1* rs150846	*BDNF* rs6265	*CIDEA* rs8092502	*PPARGC1A* rs12650562
1	201	F	168	55	19.49	18	17.9	AA	TT	CT	GA	TT	AA	GG	GA	GG	CT	CC	GA	TT	CT
2	203	F	160	45	17.58	21	2.78	AA	TT	CT	GA	TT	AA	GT	GA	GG	TT	CC	GG	CT	CT
3	204	F	167	62	22.23	18	34.1	AA	TT	CT	GA	CT	GA	TT	AA	GG	TT	CT	GG	TT	TT
4	205	F	152	48	20.78	19	10.8	AA	GT	CC	GG	CT	GA	GG	GG	GG	TT	CC	GG	TT	CC
5	206	F	155	51	21.23	19	3	GA	GT	CT	GA	CT	AA	GG	AA	GG	TT	CC	GG	CT	CT
6	207	F	165	65	23.88	18	46.5	GA	TT	CT	GG	CT	GA	GT	GA	AA	TT	CT	GG	CT	CT
7	208	F	160	56	21.88	18	46	GA	TT	CC	GG	TT	AA	GT	AA	GG	TT	CT	GG	TT	CC
8	210	F	168	61	21.61	17	18	GA	GT	CT	GA	CT	GA	GG	GA	GG	TT	TT	GG	CT	CT
9	211	F	173	58	19.38	19	17	GG	GT	TT	AA	CC	GA	GG	GG	GG	CT	CT	GG	CT	CT
10	213	F	176	110	35.51	18	38.7	GG	GT	TT	AA	CC	GA	GT	AA	GG	TT	CT	GG	CT	TT
11	214	F	169	55	19.26	19	11	GA	GG	CT	GA	CT	GA	TT	AA	GG	CT	CT	GA	CT	CC
12	216	F	157	55	22.31	19	31.7	AA	GG	CT	GG	CT	GA	GT	GA	GG	TT	CT	GG	TT	CC
13	217	F	165	65	23.88	18	12.8	GA	GG	TT	AA	TT	AA	GG	AA	GA	CT	CT	GG	TT	CC
14	219	F	158	60	24.03	19	25.4	GG	GG	TT	AA	TT	AA	GT	AA	GG	CT	CC	GG	TT	CT
15	220	F	160	60	23.44	19	32.9	AA	GT	CT	GA	TT	GA	GG	GA	GG	TT	TT	GA	CT	CC
16	223	F	161	57	21.99	18	31.4	AA	TT	CT	GA	TT	AA	GG	GA	GG	CT	CC	GG	TT	CC
17	226	F	159	54	21.36	21	22	AA	TT	TT	AA	TT	AA	GT	AA	GA	CC	CC	GG	TT	CC
18	227	F	174	75	24.77	19	26.6	GA	GT	CT	GA	CC	GG	GT	GA	GG	TT	CC	GG	CT	CT
19	228	F	164	51	18.96	20	9.6	AA	GT	CT	GA	TT	AA	GT	GA	GA	TT	CT	GG	CC	CT
20	232	F	158	52	20.83	18	23.2	AA	TT	CT	GA	CC	GG	GT	AA	GG	TT	TT	GA	CT	CC
21	233	F	168	48	17.01	18	10.6	AA	TT	TT	AA	CC	GA	GG	GA	GA	TT	TT	GA	CT	CT
22	234	F	160	57	22.27	19	17.7	AA	GT	TT	AA	CT	AA	GG	GG	GG	TT	CC	GG	CT	CT
23	236	F	150	45	20.00	19	15.4	GA	GT	TT	AA	TT	AA	GT	GA	GG	TT	CT	GG	TT	CT

24	238	F	151	48	21.05	19	9.19	GA	GT	CC	GG	TT	AA	GT	GA	GA	TT	CC	GG	CT	CC
25	239	F	160	53	20.70	19	7.84	AA	TT	TT	AA	CC	AA	GT	AA	GG	CT	CC	GA	TT	CC
26	240	F	165	45	16.53	19	15.9	GA	GT	CT	GA	TT	AA	TT	AA	GA	TT	CT	GA	CT	CC
27	242	F	157	43	17.44	19	32.7	GA	GG	CC	GG	CT	GA	GG	AA	GG	TT	CC	GG	TT	CC
28	243	F	160	54	21.09	19	22.6	GA	GT	CT	GA	TT	AA	GG	GA	GG	CT	CC	GA	CT	CC
29	244	F	164	57	21.19	18	19.5	GA	GT	CT	GA	TT	AA	GG	GA	GG	TT	CT	GG	CT	CT
30	245	M	173	52	17.37	19	7.57	GA	TT	CT	GA	CT	AA	TT	AA	GG	CT	CT	GG	TT	CC
31	246	F	166	64	23.23	18	10.8	GA	GG	CT	GA	CT	AA	GG	GA	GG	TT	CT	GG	CT	CT
32	247	F	154	50	21.08	19	11	AA	TT	CT	GA	CT	GA	GT	AA	GA	TT	CC	GG	TT	TT
33	249	F	157	55	22.31	18	37.5	GG	GG	CT	GA	CC	AA	GT	GA	GA	CT	CT	GG	TT	CT
34	250	F	155	54	22.48	19	17.8	GA	GT	TT	AA	TT	AA	GT	AA	GG	TT	TT	GG	TT	CT
35	251	F	168	60	21.26	20	15.1	AA	TT	CT	GA	CT	AA	GT	AA	GA	CT	TT	GG	CT	CC
36	252	F	164	56	20.82	19	9.93	GG	GG	CT	GA	TT	AA	GT	AA	GG	TT	CC	GA	TT	CC
37	254	F	164	56	20.82	19	21.6	AA	GT	CC	GG	CT	GG	GT	AA	GG	CT	CT	GA	CT	CC
38	256	F	170	58	20.07	18	28	GA	GT	CT	AA	TT	AA	GT	GA	GA	TT	CC	GG	CC	CT
39	257	F	156	52	21.37	19	28.1	GA	GT	CT	GG	TT	AA	GG	GA	GG	CT	CC	GG	TT	CT
40	259	M	165	49	18.00	18	6.54	GA	GT	CT	GA	TT	AA	GG	GA	GG	TT	CT	GG	TT	TT
41	260	M	170	47	16.26	18	6.12	GA	GT	CT	GA	TT	AA	GT	AA	GA	TT	CC	GA	TT	CT
42	261	F	168	54	19.13	19	8.53	GA	GT	TT	AA	TT	AA	GG	GA	GG	TT	CC	GG	CT	CT
43	262	F	161	54	20.83	19	11.9	GA	GT	TT	AA	CT	AA	GT	GA	GA	TT	CC	GG	TT	CC
44	265	F	159	54	21.36	20	5.13	AA	TT	CT	GA	TT	GA	GG	GA	GA	TT	CT	GG	TT	TT
45	266	F	156	45	18.49	18	11.3	AA	GT	CT	GA	CT	GA	GT	GA	GG	TT	CT	GG	TT	CT
46	267	F	157	44	17.85	19	13.2	GA	GT	CT	GA	CT	GA	GG	GA	GG	TT	CC	GG	CT	CT
47	268	F	155	48	19.98	19	15.8	AA	TT	TT	AA	TT	AA	GT	GA	GA	CT	CT	GG	TT	CT
48	269	F	151	37	16.23	19	14.3	AA	TT	CT	GA	CT	GA	TT	AA	GA	TT	CT	GA	TT	CC
49	270	F	151	48	21.05	19	21.1	GA	GT	CT	GG	TT	AA	GG	GG	GG	CT	CC	GG	TT	CC
50	272	F	167	57	20.44	20	57.9	GA	GT	TT	AA	TT	AA	GT	GA	GA	CT	CT	GA	CT	CT
51	274	F	150	50	22.22	19	13.2	AA	GT	TT	AA	CT	GA	GG	GA	GG	TT	TT	GA	TT	CC
52	276	F	160	59	23.05	19	19.3	GA	GT	CC	GG	CT	GA	GG	GG	GG	TT	TT	GG	TT	CT
53	277	F	170	62	21.45	17	14.1	GA	TT	CT	GG	CC	GA	TT	AA	GG	CT	CC	GA	TT	CC
54	279	F	170	68	23.53	17	13.8	GA	GT	CT	GA	CT	GA	GT	AA	GA	TT	CC	GG	TT	CT

55	281	F	157	60	24.34	18	40.1	AA	TT	CT	GA	CT	GA	GG	AA	GA	TT	CC	GG	CC	CC
56	284	F	158	55	22.03	18	21.2	AA	TT	CT	GA	CC	GG	GT	GA	GA	CT	CT	GG	CC	CC
57	285	F	157	67	27.18	20	47.5	GG	GT	TT	AA	TT	AA	GG	AA	GG	TT	CT	GG	CC	CT
58	286	M	172	84	28.39	24	10.5	AA	GT	TT	AA	CT	GA	GT	AA	GG	CT	CC	GG	TT	CT
59	287	F	173	74	24.73	17	13.6	GG	GT	CC	GG	CT	GA	GG	AA	GG	TT	CT	GA	CT	TT
60	290	F	164	80	29.74	20	57.4	AA	GT	CC	GG	CC	GG	GG	GA	GG	CT	CT	GG	TT	CC
61	292	F	157	47	19.07	19	7.29	GA	GT	CC	GG	CC	GG	TT	AA	GG	CT	CT	GA	CT	TT
62	293	M	175	90	29.39	19	62.2	AA	TT	CT	GA	CC	GA	GT	AA	GG	CT	CT	GG	TT	CT
63	294	F	165	52	19.10	24	10.2	AA	TT	TT	AA	CT	AA	GT	GA	GA	TT	CT	GA	CT	CC
64	295	F	160	64	25.00	23	21.6	GA	TT	CC	GG	CT	GA	GT	AA	GA	CT	CT	AA	CC	TT
65	296	F	161	60	23.15	18	11.5	GA	GT	CC	GG	CT	GA	GG	GA	GG	CT	CC	GA	TT	CT
66	297	F	160	56	21.88	18	20.3	AA	TT	TT	AA	TT	AA	GG	AA	GG	TT	TT	GG	CT	CC
67	298	M	166	70	25.40	20	25.7	GA	TT	TT	AA	TT	AA	GT	AA	GG	CT	CT	GA	CT	CC
68	299	F	168	55	19.49	19	11.8	GA	GG	TT	AA	CT	AA	GG	GG	GG	CC	CT	GG	TT	CT
69	300	F	164	60	22.31	21	21.1	GA	GG	CC	GG	CC	AA	GT	GA	GA	TT	CT	GG	TT	CT
70	302	F	164	52	19.33	20	9.54	GA	GG	CC	GG	CT	GA	TT	GA	GG	TT	CT	GG	CC	CC
71	303	F	170	60	20.76	19	15.4	GA	GT	TT	AA	CT	AA	GT	AA	GG	TT	CC	GA	CT	CC
72	306	F	168	53	18.78	23	23.7	GA	GT	TT	AA	CT	GA	GT	GA	GG	TT	CT	GG	CT	CT
73	307	F	160	59	23.05	19	18.8	GA	GT	CT	GG	CT	GA	GT	GA	GG	CT	CT	GG	CC	CT
74	308	F	171	57	19.49	19	6.95	GG	GT	CT	GG	CC	GG	GT	AA	GG	TT	CT	GA	TT	CC
75	310	F	160	47	18.36	18	10	GA	GT	CC	GG	CC	GG	GG	GA	GG	TT	CT	GG	TT	CT
76	312	F	155	55	22.89	19	12.9	GG	GG	CC	GG	CT	GA	GT	GA	GG	TT	CC	GG	CT	CT
77	313	F	165	48	17.63	19	8.53	GA	GT	TT	GA	TT	AA	GT	AA	GG	CT	CT	GG	CT	CC
78	315	F	167	58	20.80	20	17.3	GA	GT	CT	GA	CT	GA	GT	AA	GA	CT	CC	GA	TT	CT
79	318	F	152	55	23.81	23	23.8	GA	GT	CT	GA	CT	GA	GG	GG	GG	TT	CT	GG	TT	CT
80	319	M	173	63	21.05	25	1.1	GG	GG	CT	GA	CT	GA	GG	GA	GG	TT	CT	GA	TT	CC
81	321	F	156	41	16.85	23	11.8	AA	TT	CT	GA	CT	AA	GT	GA	GA	TT	CT	GA	TT	CC
82	322	M	178	61	19.25	20	5.62	GA	GT	CT	AA	CT	GA	GT	AA	GG	TT	CT	GG	TT	CT
83	323	M	170	70	24.22	19	18.4	GG	GG	CC	AA	CT	GA	GT	GA	GG	TT	CC	GG	TT	CT
84	327	M	178	63	19.88	19	1.59	AA	TT	CT	GA	CT	GA	TT	AA	GG	TT	CT	GG	TT	TT
85	329	M	173	67	22.39	19	18.4	AA	GT	TT	AA	TT	GA	GG	GA	GG	TT	CC	AA	TT	CC

86	330	M	179	72	22.47	19	1.19	GA	GT	CC	GG	CC	GA	GT	GA	GG	CT	CT	GG	CT	TT
87	331	M	165	69	25.34	21	3.16	GG	GG	CC	GG	CT	GA	GG	GA	GA	TT	CT	GA	CT	CC
88	333	M	172	67	22.65	20	3.34	AA	TT	CT	GA	CC	GG	TT	AA	AA	TT	CT	AA	CT	CT
89	334	M	173	80	26.73	23	4.08	AA	TT	CT	GA	CC	GA	GG	GA	GG	CT	CC	GG	CT	CT
90	335	M	167	62	22.23	22	1.9	GG	GG	CT	AA	TT	AA	GT	AA	GG	TT	CT	AA	TT	CC
91	336	F	164	54	20.08	19	26.7	GA	GT	CT	GA	CC	GA	GT	AA	GG	TT	CT	GG	TT	CC
92	337	M	173	73	24.39	22	2.92	AA	TT	CT	GA	CT	GA	GT	GA	GA	TT	CC	GG	TT	CC
93	338	M	176	60	19.37	21	0.9	AA	GT	CC	GG	CT	GA	TT	AA	GA	TT	CC	GG	CT	CC
94	339	F	167	49	17.57	18	6.06	GA	GT	CT	GA	CC	GG	GT	GA	GG	CT	CT	GG	TT	CT
95	340	F	155	50	20.81	21	6.17	GA	GT	CT	GA	CT	GA	TT	AA	GG	TT	CT	GA	CT	CT
96	342	F	157	50	20.28	20	7.93	GA	GT	CC	GG	CT	GA	GG	GA	GA	TT	CC	GG	CT	CT
97	343	F	154	51	21.50	20	4.24	GA	GT	TT	AA	TT	AA	GT	GA	GA	CT	CT	GA	CT	CT
98	344	M	180	65	20.06	22	0.84	GA	TT	CT	GA	CT	AA	GT	AA	GA	TT	CC	GG	TT	CC
99	346	M	182	66	19.93	18	0.84	GA	GT	CT	GA	CT	AA	GT	GA	GG	TT	CC	GG	TT	CT
100	347	F	161	70	27.01	19	25.4	GA	GT	CT	GA	CC	GG	GT	GA	GG	CT	CT	GG	CT	CC
101	348	M	171	68	23.26	23	2.97	GA	GG	CC	GG	TT	AA	GT	GA	GA	CT	CC	GG	CC	CC
102	349	M	171	79	27.02	19	19.5	GA	GG	CT	GA	CC	GA	GG	GA	GG	CT	CT	GG	TT	CC
103	350	M	176	60	19.37	18	1.21	AA	TT	CC	GG	CT	GA	GT	GA	GG	TT	CT	AA	TT	CT
104	351	F	171	54	18.47	18	9.5	AA	TT	TT	AA	TT	AA	GG	GA	GG	CT	CT	GG	TT	CC
105	352	F	158	57	22.83	19	12.8	AA	GT	CC	GG	CT	GA	GT	GA	GG	TT	CC	GG	CT	CC
106	353	F	160	48	18.75	19	15.3	AA	TT	CT	GA	CT	AA	GT	GA	GG	TT	TT	GA	CT	CC
107	354	M	166	54	19.60	19	1.48	GA	TT	CT	GA	CT	GA	GT	GA	GG	CT	CC	GA	TT	CT
108	357	M	171	65	22.23	20	2.19	AA	TT	CT	GG	CC	GG	GG	GA	GG	TT	CC	GG	TT	CC
109	359	M	175	75	24.49	18	18.6	AA	TT	TT	AA	TT	AA	GT	GA	GG	CT	CT	GA	CT	CC
110	361	F	160	58	22.66	18	9.06	AA	GT	TT	AA	TT	AA	GG	GA	GG	TT	CC	GG	TT	CC
111	362	M	175	80	26.12	21	0.87	GA	GT	CC	GG	TT	AA	GG	GA	GG	TT	CT	GG	TT	CT
112	363	M	187	89	25.45	21	12.6	GA	GT	TT	AA	CC	GA	GG	GA	GG	TT	CT	GA	TT	CC
113	364	M	174	55	18.17	18	6.57	AA	TT	TT	AA	CT	AA	GT	GA	GG	CT	CT	GG	TT	CC
114	365	M	171	55	18.81	19	1.62	AA	TT	CT	GA	CT	GA	GT	AA	GG	TT	CT	GG	CT	CT
115	367	F	150	47	20.89	19	14.2	GA	TT	CT	GA	TT	AA	GG	GA	GA	CT	CT	GG	TT	CC
116	368	M	175	69	22.53	20	3.98	GG	GG	CT	GA	CT	GA	GT	GA	GG	TT	CC	GA	TT	CT

117	369	F	160	53	20.70	19	7.24	GA	GT	CC	GG	CC	GA	GG	GA	GA	CT	CT	GG	TT	CT
118	370	M	174	58	19.16	22	3.74	GA	GT	CT	GA	CC	GG	GT	AA	GG	TT	CT	GA	TT	CC
119	374	M	180	70	21.60	19	5.72	GG	GG	CC	GG	CT	GA	GT	GA	AA	CT	CC	GG	TT	CT
120	375	F	165	70	25.71	17	47.9	AA	TT	TT	AA	CT	AA	GT	GA	GG	TT	CT	GA	TT	CC
121	376	F	160	55	21.48	19	17.8	AA	TT	CT	GA	TT	AA	GG	GG	GG	TT	CT	GG	TT	CC
122	378	F	155	50	20.81	17	32.2	GG	GG	CC	GG	CC	GG	GT	GA	GA	TT	CC	GG	CT	CT
123	379	F	162	47	17.91	18	6.53	GA	TT	CT	GA	TT	AA	GT	GA	GG	TT	CC	GG	TT	TT
124	380	M	175	72	23.51	18	8.63	GA	GG	CT	GA	CT	GA	GT	GA	GG	CT	CT	GG	CT	CC
125	381	M	175	60	19.59	20	0.9	GA	GT	CC	GG	CT	GA	TT	AA	GG	CT	CC	GG	CT	CC
126	382	M	170	52	17.99	18	3.62	GA	TT	CT	GA	TT	AA	GT	AA	GG	CT	CC	GG	CT	CC
127	383	M	170	68	23.53	23	2.66	AA	TT	TT	AA	TT	AA	TT	AA	GG	CT	CT	GA	TT	CT
128	384	M	165	65	23.88	22	5.61	AA	TT	CC	GG	TT	AA	GG	GA	GA	TT	CC	GG	TT	CC
129	386	M	163	61	22.96	21	3.6	GA	GT	CT	GA	TT	AA	GG	GA	GG	TT	CC	GA	TT	TT
130	387	F	169	56	19.61	18	10.1	GA	GT	CT	GA	TT	AA	TT	AA	GA	TT	CC	GG	TT	CC
131	389	M	170	68	23.53	21	1.15	AA	TT	TT	AA	CC	GA	GT	GA	GG	TT	CC	GG	TT	TT
132	390	M	182	81	24.45	22	1.56	AA	TT	TT	AA	TT	AA	GG	GG	GA	TT	CC	GG	CT	TT
133	391	M	184	84	24.81	21	1.38	GG	GT	CT	GA	CT	GA	GG	GA	GG	TT	CC	GA	TT	TT
134	392	F	170	75	25.95	18	27.1	AA	TT	TT	AA	TT	AA	GG	GA	GG	CT	CC	GG	TT	CC
135	393	M	170	69	23.88	20	2.99	GA	GT	CC	GG	CC	GA	GG	GA	GG	TT	CT	GA	TT	TT
136	394	M	171	62	21.20	27	7.55	GA	GT	CC	GG	CT	GA	TT	AA	GG	TT	CC	GG	TT	CT
137	395	F	163	48	18.07	18	7.42	AA	GT	TT	AA	TT	AA	GT	GA	GG	CT	TT	GG	TT	CT
138	396	M	175	68	22.20	19	1.05	AA	TT	CC	GG	CT	GG	GG	AA	GA	CT	CC	GA	TT	TT
139	397	M	167	58	20.80	19	1.56	GA	GT	CC	GG	CT	GA	GT	GA	GA	TT	CC	GG	TT	CC
140	398	F	164	68	25.28	21	25	GA	GT	CT	GA	CT	GA	TT	AA	GA	CT	CT	GA	TT	CT
141	399	M	169	70	24.51	19	12.1	GA	GT	CT	GA	CT	GA	GG	GA	GG	CT	CC	GG	CT	CC
142	400	M	170	55	19.03	24	3.72	GA	GT	TT	AA	CC	GA	TT	AA	GA	CT	CC	GG	TT	CC
143	402	M	180	68	20.99	22	13.3	GA	GT	CT	GA	CT	AA	GT	GA	GG	TT	CC	GG	TT	CT
144	403	M	178	72	22.72	22	0.73	AA	TT	TT	AA	CC	AA	GT	AA	GG	TT	CC	GA	TT	CT
145	404	M	180	75	23.15	19	9.06	GA	GT	TT	AA	CT	AA	GG	GG	GA	CT	CC	GG	TT	CT
146	406	M	168	58	20.55	19	1.36	AA	TT	CC	GG	CC	GG	GG	GA	GG	CC	CT	GA	TT	CT
147	407	M	172	72	24.34	18	2.3	GA	GG	CT	GA	CC	GG	GT	GA	GG	CT	CT	GA	TT	CT

148	408	M	161	55	21.22	18	2.31	AA	TT	TT	AA	CT	GA	GG	GG	GG	TT	CT	AA	TT	CC
149	410	F	157	42	17.04	19	9.54	AA	GT	CT	GA	TT	AA	GT	GA	GG	TT	CT	GA	TT	CT
150	412	M	175	67	21.88	18	4.33	AA	TT	CT	GA	CC	GA	GT	GA	GG	CC	TT	AA	CT	CC
151	413	F	158	45	18.03	18	5.02	AA	TT	CT	GG	TT	AA	GT	GA	GG	TT	CT	GG	TT	CT
152	414	M	175	63	20.57	17	3.51	AA	TT	CT	GA	TT	AA	GT	AA	GG	TT	CT	GA	TT	CC
153	416	M	169	54	18.91	19	1.38	AA	TT	CC	GA	CT	GA	GT	AA	GG	TT	CT	GA	TT	CC
154	417	F	155	42	17.48	18	15.4	GA	TT	CT	GG	CT	GA	TT	GG	GG	TT	CC	GG	CT	CC
155	418	M	172	58	19.61	18	2.05	AA	GT	CC	GG	CT	GA	GG	AA	GG	TT	CC	GG	TT	CC
156	419	M	173	80	26.73	19	11.2	GA	GT	CC	GG	CT	GA	TT	AA	GG	CT	CC	GG	TT	CT
157	603	F	162	53	20.20	19	12.5	GG	GT	CC	GG	CT	AA	GG	GG	GA	TT	TT	GG	TT	CC
158	604	M	160	44	17.19	19	8.61	AA	TT	CT	GA	CT	AA	GT	GA	GG	TT	CC	GG	CT	CT
159	605	F	153	50	21.36	19	12.3	GG	GG	CT	GA	TT	GA	GT	AA	GG	CC	CC	GG	CT	TT
160	606	M	170	71	24.57	20	0.95	GG	TT	TT	AA	TT	AA	GG	GA	GG	TT	CC	GA	TT	CC
161	607	F	160	61	23.83	19	17.7	AA	TT	CT	GA	CT	GA	GG	GA	GG	CT	CT	GG	TT	CT
162	608	M	186	65	18.79	18	1.95	GG	GT	TT	AA	CT	GA	GT	AA	AA	CT	CC	GG	CT	TT
163	609	M	175	75	24.49	19	13.8	AA	GT	TT	AA	TT	AA	GT	AA	GA	TT	CT	GG	TT	CT
164	611	F	160	59	23.05	19	28.7	AA	TT	CT	GA	CT	GA	GT	GA	GG	CT	CT	GG	CT	CC
165	612	F	164	52	19.33	19	20.9	GA	GG	TT	AA	CT	GA	GT	GA	GG	TT	CT	GG	TT	CC
166	613	F	161	63	24.30	19	27.7	GA	TT	CC	GG	CT	AA	TT	GA	GA	TT	CC	GG	TT	CT
167	614	F	151	56	24.56	19	31.3	AA	TT	CT	GA	TT	AA	GG	AA	GG	TT	CC	GA	TT	CT
168	615	M	172	70	23.66	19	4.13	GG	GT	CT	GA	CT	GA	GG	GA	GA	TT	CC	GG	TT	CC
169	617	F	160	56	21.88	18	69.1	GG	GT	CT	GA	CC	GG	GG	GA	GG	TT	CT	GG	CT	CC
170	618	M	178	75	23.67	22	10.6	GG	TT	CT	GA	CT	GA	GG	GA	GA	CT	CT	GG	TT	CT
171	619	F	165	56	20.57	19	10.7	AA	GT	CC	GG	CC	GA	GG	GA	GG	TT	CT	GA	CT	CC
172	620	F	156	45	18.49	18	14.1	GG	GT	CC	GG	CC	GG	GT	AA	GG	CT	TT	GG	CT	CC
173	621	M	177	49	15.64	20	5.43	GA	GT	CT	GA	TT	AA	GT	GA	GG	CT	CT	GG	CT	TT
174	622	F	157	65	26.37	19	42	GG	GT	TT	AA	CT	GA	GT	AA	GG	TT	CT	GG	TT	CC
175	623	F	155	52	21.64	21	21.9	GA	GT	CC	GG	CT	GA	GT	AA	GG	CT	CC	GA	CT	CT
176	624	F	160	60	23.44	18	10	GA	TT	CC	GG	CC	GA	GT	GA	GG	CT	CC	GG	CT	CT
177	625	F	160	54	21.09	19	21.2	AA	TT	CC	GG	CC	GG	GT	GA	GG	CT	CT	GG	TT	TT
178	627	M	185	100	29.22	19	22.9	GA	TT	CT	GA	CT	AA	GT	GA	GG	TT	CT	GG	TT	CT

179	629	M	162	48	18.29	20	4.47	AA	TT	CC	GG	CT	GA	GT	GA	GG	TT	CC	GG	CT	CC
180	630	M	176	51	16.46	19	4.7	GA	GT	CT	GA	TT	AA	GG	GA	GA	TT	CT	GA	TT	CT
181	631	M	174	68	22.46	25	4.2	AA	GT	CC	GG	CC	GG	GG	GG	GA	CT	CC	GG	CT	CT
182	632	F	155	53	22.06	29	8.83	AA	GT	CT	GA	TT	AA	GT	GA	GG	TT	CT	GG	CC	CC
183	633	F	160	67	26.17	19	11.7	GG	GT	TT	GA	TT	AA	GT	AA	GG	CT	CC	GG	CT	CC
184	635	F	160	55	21.48	19	13.9	GA	GT	CC	GG	CC	GG	GG	AA	GA	TT	CC	GG	TT	CT
185	636	M	188	80	22.63	19	5.39	GA	GT	CT	GA	TT	AA	TT	AA	GG	CT	CC	GG	TT	CC
186	637	M	175	63	20.57	19	1.46	GA	GT	TT	AA	TT	AA	GT	GA	GG	CC	CC	GG	TT	CC
187	638	F	172	60	20.28	19	11	AA	TT	CT	GA	CC	GA	TT	AA	GG	CT	CT	GA	CT	CC
188	639	F	158	49	19.63	19	15.9	AA	TT	CT	GA	TT	AA	GT	GA	GG	TT	CT	GA	TT	CT
189	640	F	150	56	24.89	19	37	AA	TT	TT	AA	TT	AA	GG	GA	GG	TT	CT	GG	TT	CT
190	641	M	183	84	25.08	19	12.3	GG	GG	TT	AA	CC	AA	GT	GA	GA	TT	CC	GA	CC	CT
191	642	M	179	65	20.29	18	1.35	AA	TT	TT	AA	TT	AA	GT	GA	GG	TT	CT	GG	CT	CT
192	644	M	170	53	18.34	19	4.47	GG	GG	CT	GA	CC	GA	GT	GA	GG	CT	CT	GG	TT	CT
193	646	F	163	60	22.58	19	23.3	GG	GG	CT	GA	TT	AA	TT	AA	GG	TT	CC	GG	TT	CC
194	647	M	182	77	23.25	19	10.8	AA	GT	TT	AA	CT	AA	GG	GA	GG	CT	CT	GG	CT	CT
195	648	F	155	38	15.82	19	4.25	GA	GT	TT	AA	TT	AA	GG	GA	GA	TT	CC	GG	TT	CC
196	649	M	173	78	26.06	20	6	GA	GT	TT	AA	TT	AA	GT	AA	GG	CT	CT	GG	TT	CT
197	650	F	150	45	20.00	21	10.6	GG	GG	TT	AA	TT	AA	GG	GG	GG	TT	CT	GA	TT	CC
198	651	M	171	60	20.52	18	32.3	GA	GT	CT	GA	CC	GG	GG	AA	GA	CT	CT	GG	TT	TT
199	652	F	165	58	21.30	22	37.1	AA	TT	CC	GG	CT	GA	GT	AA	GG	CT	CT	GG	CT	TT
200	653	F	160	47	18.36	19	19.9	GA	GT	TT	AA	TT	AA	GT	AA	GG	CT	CT	GG	TT	TT
201	655	F	155	50	20.81	19	17.4	GA	TT	CC	GG	CT	GA	GT	AA	GA	CT	CC	GG	TT	CT
202	656	F	167	58	20.80	19	5.79	AA	GT	CC	GG	CC	GG	GT	GA	GG	CC	CC	GA	TT	TT
203	657	F	164	63	23.42	19	20.4	AA	GT	TT	AA	CT	AA	GT	AA	GA	TT	TT	GG	TT	CT
204	658	F	163	63	23.71	19	19.3	GA	GT	TT	AA	CT	AA	TT	AA	GG	CT	CT	GG	TT	CC
205	659	F	161	60	23.15	19	53.2	AA	TT	CT	GA	CT	GA	GG	GA	GG	TT	CT	GG	TT	CT
206	660	F	168	69	24.45	19	23.4	GG	TT	TT	AA	CC	GG	GG	AA	GG	TT	CC	GG	TT	CT
207	661	F	162	44	16.77	19	19.2	AA	TT	CT	GA	TT	AA	GG	GA	GA	CT	CC	GA	TT	CC
208	662	M	176	75	24.21	18	5.06	AA	TT	CT	GA	CT	AA	GT	AA	GG	TT	CC	GG	CT	CC
209	663	M	164	50	18.59	22	1.51	AA	GG	TT	AA	TT	AA	GG	GG	GG	CC	CT	GG	TT	CT

210	664	F	161	59	22.76	19	40.1	AA	TT	CC	GG	CT	GA	GG	GA	GG	CT	CT	GG	CT	CC
211	675	F	165	60	22.04	19	7.88	AA	TT	CT	GA	CT	AA	GT	GA	GA	CT	CC	GG	CT	CT
212	678	F	162	52	19.81	18	12.2	GA	GT	CT	GA	CC	GG	GG	GG	GG	TT	CT	GA	TT	CC
213	681	F	160	51	19.92	25	4.71	GA	TT	CT	GA	CT	GA	GG	GA	GG	TT	CT	GA	CT	CT
214	682	F	157	48	19.47	23	12.3	GA	GT	CC	GG	CT	GA	GG	GA	GG	CT	CT	GG	TT	CC
215	683	F	165	54	19.83	30	21.3	GG	GG	TT	AA	TT	AA	GG	AA	GG	CT	CT	GG	CT	CC
216	684	F	165	59	21.67	23	13.8	GA	GT	TT	AA	CT	AA	GG	AA	GA	TT	CT	GG	TT	CC
217	686	F	164	62	23.05	20	22.5	GA	GG	TT	AA	CT	GA	TT	AA	GG	CC	CC	GG	TT	TT
218	687	F	167	58	20.80	19	14.3	GA	TT	TT	AA	TT	AA	GT	GA	GG	CT	CC	GG	CT	TT
219	689	F	158	54	21.63	19	20.6	GA	GT	CT	GA	CT	GA	TT	AA	GA	TT	TT	GG	TT	CC
220	690	F	158	54	21.63	19	14.5	AA	GT	CT	GA	TT	AA	GT	GA	GG	CT	CC	GG	CT	CT
221	691	M	180	66	20.37	18	1.9	GA	GT	CT	GA	CC	GA	GT	GA	GG	TT	CC	GG	TT	TT
222	692	F	164	53	19.71	20	21.2	AA	TT	TT	AA	TT	AA	GG	GA	GG	TT	CC	GG	CT	CC
223	693	M	173	76	25.39	20	25	AA	TT	CC	GG	CT	GA	GG	AA	GG	CT	CC	GA	CT	TT
224	694	M	165	65	23.88	18	2.54	AA	TT	TT	AA	CC	GA	GG	GA	GA	CT	CC	GA	TT	CT
225	695	F	168	59	20.90	19	37.2	GA	GG	TT	AA	CT	GA	GG	GA	GG	CT	CC	GG	TT	CC
226	696	F	167	65	23.31	20	13.3	GA	GT	CC	GG	CC	GG	GG	GA	GA	TT	CC	GG	CT	CT
227	712	M	173	74	24.73	18	6.43	GG	GG	TT	AA	TT	AA	GG	GG	GG	CC	TT	GA	TT	CC
228	713	F	154	57	24.03	22	29	AA	TT	CT	GA	CT	GA	GG	GG	GA	TT	CT	GA	TT	CC
229	715	F	167	55	19.72	19	11.3	AA	GT	CT	GA	TT	AA	GT	AA	GA	TT	CT	GA	TT	CT
230	716	F	160	60	23.44	19	10.7	GA	GT	CT	GA	CC	GA	GG	GG	GA	TT	CT	GA	TT	CC
231	717	M	156	50	20.55	20	3.95	GA	TT	CC	GG	CT	GA	GG	AA	GG	TT	CT	GG	TT	CC
232	719	F	153	46	19.65	21	7.86	GG	GG	CT	GA	CT	GA	GT	GA	GG	TT	CC	GG	CT	CC
233	720	F	162	52	19.81	20	6.44	AA	TT	CT	GA	CC	GG	TT	AA	GG	CT	CT	GA	TT	TT
234	721	M	160	50	19.53	20	1.56	GG	GG	CT	GA	TT	AA	GT	AA	GG	TT	CC	GA	CT	CT
235	722	F	160	66	25.78	23	62.1	AA	TT	CC	GG	CT	AA	GG	GG	GG	TT	CC	GA	CT	TT
236	724	F	165	66	24.24	23	26.3	GG	GG	CC	GG	TT	AA	GT	GA	GG	TT	CT	GG	TT	CC
237	725	F	163	50	18.82	24	7.42	GA	GG	CT	GA	CC	GG	GG	GA	GG	CT	CT	GG	TT	CT
238	726	F	163	60	22.58	22	15.8	GA	GT	CT	GA	CC	GA	GT	AA	GG	CT	CC	GG	TT	CC
239	727	F	173	65	21.72	24	12.2	GA	GG	CT	GA	CT	GA	GT	GA	GG	TT	TT	GG	CT	CT
240	728	F	156	52	21.37	28	9.45	AA	GT	CT	GA	TT	AA	GG	GG	GG	TT	CC	GG	CT	TT

241	729	F	160	43	16.80	18	18.5	GA	GT	CT	GA	CC	GG	GT	GA	GA	CT	CT	GA	TT	CC
242	730	F	171	66	22.57	19	28.4	AA	GT	CT	GA	CC	GA	GG	AA	GG	TT	CC	GA	TT	CT
243	732	F	168	60	21.26	19	36.3	AA	TT	CC	GG	CC	AA	GG	GA	GA	TT	CC	GG	TT	CT
244	733	F	157	60	24.34	23	8.22	AA	GT	CC	GG	CT	GA	GG	GG	GG	TT	CT	GA	CT	TT
245	735	F	163	45	16.94	19	11	GG	GG	CT	GA	CT	AA	GT	AA	GA	TT	CC	GA	TT	CT
246	736	F	158	55	22.03	19	11.7	GA	GT	CC	GG	CC	GG	GG	AA	AA	TT	CC	GA	TT	CC
247	737	M	172	62	20.96	17	1.92	GG	GT	CT	GA	CT	GA	GT	GA	GG	TT	CT	GA	TT	CT
248	738	F	150	42	18.67	24	18.4	AA	GT	CC	GG	CT	AA	GT	AA	GG	CT	CT	GG	CT	TT
249	739	F	160	75	29.30	24	55.6	AA	TT	CC	GG	CT	GA	GG	GG	GG	TT	TT	GG	TT	CT
250	740	F	161	50	19.29	23	13.5	GA	GG	CT	GA	CT	GA	GG	GG	GG	CC	CC	GA	TT	CT
251	741	F	163	67	25.22	23	20.7	GA	GT	TT	AA	TT	AA	GT	GA	GA	TT	CT	GA	CT	CC
252	742	F	164	47	17.47	22	7.01	GA	GT	CT	GA	TT	AA	GG	AA	GG	CT	TT	GG	CT	CT
253	743	F	173	63	21.05	23	6.09	GA	GT	CC	GG	CC	GG	GT	GA	GG	CT	CC	GA	CT	CT
254	745	F	158	50	20.03	21	16.9	GG	GT	TT	AA	CT	AA	GG	GA	GG	TT	TT	GG	CT	CC
255	746	F	166	60	21.77	21	23.7	AA	GT	CC	GG	CT	GA	GT	AA	GG	CT	CT	GG	CT	CC
256	747	F	163	60	22.58	21	22.5	AA	GT	CT	GA	CT	GA	GT	AA	GG	TT	CT	GG	TT	CT
257	749	F	150	48	21.33	21	7.65	GA	GT	CT	GA	TT	AA	GT	GA	GA	TT	CC	GA	TT	CT
258	751	F	152	40	17.31	21	27.6	GA	GT	CT	GA	CT	GA	GT	AA	GG	CT	TT	GG	TT	CT
259	752	M	177	55	17.56	20	3.94	AA	TT	CT	GG	CT	GA	GT	AA	GG	CT	CT	GG	CT	CC
260	753	F	159	48	18.99	21	13.6	GA	GT	CC	GG	CT	GA	GG	AA	AA	CT	CT	GG	TT	CC
261	754	F	160	75	29.30	20	43.2	AA	TT	CT	GA	CT	AA	GG	GA	GG	CT	CT	GG	TT	CT
262	755	M	172	67	22.65	22	1.36	GA	TT	CT	GA	TT	AA	TT	AA	GG	CT	CC	GG	CT	CC
263	756	F	166	48	17.42	22	35	AA	TT	CT	GA	CT	AA	GG	GG	GG	CT	TT	GG	TT	CC
264	758	F	152	43	18.61	20	7.49	GG	GG	CT	GA	CT	AA	GT	AA	GA	TT	CC	GG	TT	CT
265	759	F	148	45	20.54	19	8.27	GA	TT	TT	AA	CT	AA	GG	GA	GG	TT	CT	GG	CT	CC
266	760	M	175	75	24.49	24	8.32	GA	GT	CT	GA	TT	AA	GG	GG	GG	CT	CT	GG	CT	CT
267	761	F	166	55	19.96	21	10.9	GG	GG	CC	GG	CC	GG	GG	AA	GG	TT	CC	GA	TT	CT
268	762	M	171	71	24.28	20	8.97	AA	TT	TT	AA	CT	AA	GT	GA	GA	CT	CT	GG	CT	CT
269	763	F	155	54	22.48	22	15.1	GA	GG	CT	GA	CT	GA	GG	GA	GA	TT	CT	GG	TT	CC
270	764	F	160	66	25.78	20	6.16	AA	TT	CC	GG	CT	GA	GT	AA	GG	TT	CC	GG	CT	CC
271	765	F	158	48	19.23	20	15.1	GA	GT	CT	GG	CC	GA	GG	AA	GG	TT	CC	GG	CC	CT

272	766	F	158	79	31.65	25	12	GA	GT	CT	GA	CC	GA	GT	GA	GG	TT	CC	GG	CT	CC
273	767	F	160	51	19.92	20	13.3	GG	GT	TT	AA	CT	AA	GT	GA	GG	CT	TT	GG	CT	CC
274	768	F	157	48	19.47	20	16	GA	GT	CC	GG	CT	GA	TT	GA	GG	CT	CT	GG	CT	CC
275	769	F	150	48	21.33	20	14.6	AA	TT	CT	GA	CC	GA	TT	AA	GA	CC	CT	GG	TT	CC
276	771	M	174	62	20.48	23	2.4	AA	TT	CC	GG	CT	GA	GT	GA	GA	TT	CT	GA	TT	CC
277	772	M	172	67	22.65	21	2.48	AA	TT	CC	GG	CC	GG	GT	GA	GG	CT	CT	GA	TT	TT
278	773	F	162	51	19.43	21	6.97	GA	GT	CT	GA	CT	GA	GG	GA	GG	CT	CC	GA	CT	CC
279	774	F	156	60	24.65	22	7.93	GA	GG	CC	GG	CC	GG	GT	AA	GG	CT	CC	GG	TT	CT
280	775	M	182	75	22.64	21	5.41	GG	GG	CT	GA	CC	GA	GT	GA	GA	CT	CC	GG	TT	CC
281	776	M	173	70	23.39	19	11.5	GA	TT	TT	AA	TT	AA	TT	AA	GG	CC	CC	AA	TT	CC

F – females; M – males.

Table S2. Allele frequencies of studied SNP-markers of *UCP1, UCP2, UCP3, FNDC5, LEPR, PTGS2, TRPV1, BDNF, CIDEA, PPARGC1A* genes in the Yakut population

No	SNP	Gene	Genotypes, n			Allele frequency		HWE[1]
1	rs1800592	*UCP1*	AA<br>95	AG<br>108	GG<br>41	A<br>0.618	G<br>0.382	0.468
2	rs3811787	*UCP1*	TT<br>86	TG<br>115	GG<br>43	T<br>0.583	G<br>0.417	0.356
3	rs659366	*UCP2*	TT<br>67	TC<br>110	CC<br>67	T<br>0.503	C<br>0.497	0.593
4	rs660339	*UCP2*	AA<br>70	AG<br>100	GG<br>74	A<br>0.473	G<br>0.527	0.05
5	rs1800849	*UCP3*	TT<br>69	TC<br>114	CC<br>61	T<br>0.538	C<br>0.462	0.155
6	rs2075577	*UCP3*	AA<br>102	AG<br>111	GG<br>31	A<br>0.659	G<br>0.341	0.499
7	rs1137101	*LEPR*	AA<br>6	AG<br>65	GG<br>173	A<br>0.156	G<br>0.844	0.519
8	rs16835198	*FNDC5*	TT<br>28	TG<br>115	GG<br>101	T<br>0.341	G<br>0.659	0.356
9	rs3480	*FNDC5*	AA<br>94	AG<br>125	GG<br>25	A<br>0.637	G<br>0.363	0.468
10	rs689466	*PTGS2*	TT<br>140	TC<br>91	CC<br>13	T<br>0.777	C<br>0.223	0.356
11	rs150846	*TRPV1*	AA<br>18	AG<br>46	GG<br>110	A<br>0.360	G<br>0.640	0.115
12	rs6265	*BDNF*	AA<br>8	AG<br>73	GG<br>163	A<br>0.151	G<br>0.849	0.468
13	rs8092502	*CIDEA*	TT<br>144	TC<br>88	CC<br>12	T<br>0.750	C<br>0.250	0.512
14	rs12650562	*PPARGC1A*	TT<br>31	TC<br>102	CC<br>111	T<br>0.320	C<br>0.680	0.969

[1] – p-values of deviation from Hardy–Weinberg Equilibrium (HWE).

Table S3. ANOVA analysis of serum leptin levels depending on genotypes in the Yakuts with normal weight (n=215)

Gene, SNP	Mean±std.dev			F; p
UCP1, rs1800592	GG	AG	AA	
F	19.59±14.09	16.41±9.88	19.97±10.79	F=1.8057; p=0.16814
M	4.67±4.79	5.71±6.6	4.24±4.83	F=0.50827; p=0.60380
UCP1, rs3811787	TT	GT	GG	
**F**	**21.94±12.28**	**16.31±10.33**	**17.27±9.19**	**F=3.8329; p=0.02394**
M	3.57±3.91	6.25±6.93	4.99±4.83	F=1.7959; p=0.17377
UCP2, rs659366	CC	CT	TT	
F	17.08±10.07	18.77±11.96	18.24±9.98	F=0.29506; p=0.74495
M	3.57±4.06	4.95±5.97	5.97±5.9	F=0.93174; p=0.39884
UCP2, rs660339	AA	AG	GG	
F	18.05±10.58	18.09±11.94	18.5±9.97	F=0.02142; p=0.97882
M	2.79±1.92	4.99±6.15	6.32±6.144	F=2.1595; p=0.12323
UCP3, rs1800849	CC	TC	CC	
F	17.45±12.58	18.05±10.13	18.9±11.02	F=0.19620; p=0.82207
M	4.45±7.29	4.63±4.43	5.62±5.64	F=0.26135; p=0.77078
UCP3, rs2075577	GG	GA	AA	
F	17.42±14.52	18.72±10.64	17.9±10.12	F=0.14249; p=0.86732
M	5.88±9.96	4.26±4.49	5.32±4.87	F=0.45380; p=0.63712
LEPR, rs1137101	GG	GA	AA	
F	18.31±10.74	17.44±11.04	23.93±19.57	F=0.509; p=0.602
M	4.49±4.8	6.03±7.32	3.67±1.9	F=0.60242; p=0.55038
FNDC5, rs3480	AA	GA	AA	
F	17.64±8.82	19.21±13.04	15.34±5.8	F=0.92780; p=0.39782
M	4.61±6.5	5.06±5.19	4.77±3.19	F=0.05015; p=0.95113
FNDC5, rs16835198	GG	TG	TT	
F	18.5±12.01	18.4±10.55	15.86±8.11	F=0.40086; p=0.67050
M	6.02±7.01	4.34±4.72	3.89±3.41	F=0.86918; p=0.42390
PTGS2, rs689466	TT	TC	CC	
F	18.48±11.63	18.15±10.38	14.64±5.9	F=0.39306; p=0.67572
M	3.86±4.44	6.58±6.93	4.43±4.02	F=1.9693; p=0.14744
TRPV1, rs150846	GG	GA	AA	
F	16.61±8.84	19.62±13.28	18.07±5.3	F=1.2130; p=0.30039
M	4.39±4.61	5.45±6.67	5.38±1.48	F=0.31406; p=0.73153
BDNF, rs6265	GG	GA	AA	
**F**	**19.64±11.05**	**14.65±10.01**	**ND**	**F=6.4003; p=0.01250**
M	5.58±6	2.1±3.7	6.14±6.41	F=1.7157; p=0.18753
CIDEA, rs8092502	CC	TC	TT	
F	18.9±10.87	17.73±12.6	18.44±9.72	F=0.08568; p=0.91794
M	2.97	5.09±4.89	4.82±5.79	F=0.07504; p=0.92778
PPARGC1a, rs12650562	TT	TC	CC	
F	15.12±9.92	18.57±11.37	18.5±10.84	F=0.64929; p=0.52397
M	4.43±8.81	5.36±4.78	4.62±4.6	F=0.17608; p=0.83893

F – females; M – males; ND – no data.

Table S4. ANOVA analysis of BMI, weight, and height depending on genotypes in the females with normal weight (n=144)

Gene, SNP	Mean±std.dev			F; p
UCP1, rs3811787	TT	TG	GG	
BMI	21.7±1.62	21.3±1.53	21.33±1.84	F=0.88198; p=0.41623
Weight	55.79±5.24	55.41±6.06	55.46±6.12	F=0.05971; p=0.94206
Higher	160.33±5.75	161.16±6.26	161.18±5.96	F=0.29205; p=0.74718
BDNF, rs6265	GG	GA	AA	
**BMI**	**21.57±1.6**	**21.02±1.6**	**ND**	**F=3.67986; p=0.05**
Weight	55.68±5.94	55.19±5.52	ND	F=0.20758; p=0.64937
Higher	160.47±5.77	162±6.56	ND	F=1.92866; p=0.16708

ND – no data.

Table S5. Prevalence of the T-allele of rs3811787 (*UCP1*) in eight East Asian populations living in different climatic zones

Populations	n	Climatic zones	Prevalence of the T allele (CI)	Total prevalence of the T allele (CI)	p
"North Asia"					
Northern Yakuts (N.YAK)	16	subarctic	0.72 (0.491-0.877)	0.63 (0.546-0.652)	0.02
Vilyuy Yakuts (V.YAK)	67	temperate	0.55 (0.441-0.659)		
Central Yakuts (C.YAK)	198	temperate	0.61 (0.542-0.667)		
"South Asia"					
Chinese (CHB)	103	temperate	0.51 (0.421-0.598)	0.49 (0.448-0.529)	
Japanese (JPT)	104	subtropical	0.56 (0.473-0.648)		
Chinese (CHS)	105	subtropical	0.49 (0.403-0.579)		
Chinese (CDX)	93	subequatorial	0.46 (0.365-0.551)		
Vietnamese (KHV)	99	subequatorial	0.41 (0.327-0.506)		
Total			n=785		

n – number of individuals; CI – confidence interval; "North Asia": N.YAK – Northern Yakuts, V.YAK – Vilyuy Yakuts, C.YAK – Central Yakuts; "South Asia": CHB – Han Chinese, JPT – Japanese, CHS – Southern Han Chinese, CDX – Chinese Dai, KHV – Vietnamese.

Table S6. Prevalence of the G-allele of rs6265 (*BDNF*) in eight East Asian populations living in different climatic zones

Populations	n	Climatic zones	Prevalence of the G allele (CI)	Total prevalence of the G allele (CI)	p
"North Asia"					
Northern Yakuts (N.YAK)	16	subarctic	0.84 (0.627-0.951)	0.83 (0.779-0.862)	0.01
Vilyuy Yakuts (V.YAK)	67	temperate	0.84 (0.766-0.921)		
Central Yakuts (C.YAK)	198	temperate	0.81 (0.756-0.857)		
"South Asia"					
Chinese (CHB)	103	temperate	0.50 (0.407-0.584)	0.51 (0.471-0.552)	
Japanese (JPT)	104	subtropical	0.61 (0.521-0.694)		
Chinese (CHS)	105	subtropical	0.47 (0.384-0.56)		
Chinese (CDX)	93	subequatorial	0.52 (0.422-0.609)		
Vietnamese (KHV)	99	subequatorial	0.46 (0.375-0.556)		
Total			n=785		

n – number of individuals; CI – confidence interval; "North Asia": N.YAK – Northern Yakuts, V.YAK – Vilyuy Yakuts, C.YAK - Central Yakuts; "South Asia": CHB – Han Chinese, JPT – Japanese, CHS – Southern Han Chinese, CDX – Chinese Dai, KHV – Vietnamese.

Table S7. List of primers sequences, annealing temperature and allelic profiles of the studied SNPs

Gene	Chromosomal region	SNP	Primer sequence	Annealing temperature/ time, product size	Restriction enzymes, allele sizes (bp)
UCP1	4q31.1	rs1800592<br>-3826A>G	F: 5'-ACATTTTGTGCAGCGATTTCTG-3'<br>R: 5'-TTCACCACTTCTGACAGGCT-3'	56°C/ 45 sec., 301 bp	*Ksp22I*<br>A = 265+36<br>G = 301
		rs3811787<br>-412A>C	F: 5'-CCTTCTGTCACCCTTTGGCTGCACACCTTCGCC-3'<br>R: 5'- TGACAAGTTCAGAGTGCTCTT-3'	57°C/ 45 sec., 296 bp	*Bst2UI*<br>T = 262+34<br>G = 296
UCP2	11q13.4	rs659366<br>−866G>A	F: 5'-AGCGTGACCTCACGCTCCTA-3'<br>R: 5'-GACTGAACGTCTTTGGGACTCCGT-3'	59°C/ 45 sec., 299 bp	*BspFNI*<br>T = 178+121<br>C = 299
		rs660339<br>Ala55Val	F: 5'-TTGACAGAATCATACAGGCCGA-3'<br>R: 5'-TTGGAGCATCGAGATGACTG-3'	53,8°C/ 45 sec., 392 bp	*Bst4CI*<br>G = 392<br>A = 153+110
UCP3	11q13.4	rs1800849<br>-55C>T	F: 5'-CCTTGTCACCAAGGAAGCGTCCACAGCTT-3'<br>R: 5'-CTTCTGGCTTGGCACTGGTCTTATACACCC-3'	59°C/ 45 sec., 215 bp	*SmaI*<br>C = 185+30<br>T = 215
		rs2075577<br>Tyr210Tyr	F: 5'-GGGACTGGAACCCAAGTCT-3'<br>R: 5'-ACGACATCCTCAAGGAGAAGCTGCTGGAGTA-3'	58°C/ 45 sec., 249 bp	*RsaNI*<br>G = 218+32<br>A = 249
LEPR	1p31.3	rs1137101<br>Gln223Arg	F: 5'-CAGTTTTCAGATGGTTCACTGC-3'<br>R: 5'-TCTCTGACAAACATGAGCCTC-3'	55°C/ 45 sec., 539 bp	*HpaII*<br>A = 539<br>G = 409+130
FNDC5	1p35.1	rs16835198<br>G>T	F: 5'-TTGCCTCATAGAAACACTCACCAGTCTG-3'<br>R: 5'-TAATCACCTAACACCTCAGGCAAGTGAATT-3'	57°C/ 45 sec., 203 bp	*EcoRI*<br>G = 170+33<br>T = 203
		rs3480<br>A>G	F: 5'-CTCTTGTAGACCGGAAGGAAGGGCCGGTCATT-3'<br>R:5'-GAGCAGCTCTGGCTATCAC-3'	55°C/ 45 sec., 424 bp	Bsc4I<br>G = 380+34<br>A = 424
PTGS2	1q31.1	rs689466<br>−1195G>A	F: 5'-ATGAGTTGTGACCATGGATCAA-3'<br>R: 5'-AAAAACCTCCAAGTGAGTCTCTT-3'	58°C/ 45 sec., 432 bp	*Bst4CI*<br>T = 432<br>C = 295+137
TRPV1	17p13.2	rs150846<br>−6355A>C	F: 5'-CACCTTTGAACTCGTTGTC-3'<br>R: 5'-GTCTGGGAGTAGGAGGGTGACTTTCTAGATCG-3'	57,5°C/ 45 sec., 424 bp	*BspFNI*<br>G = 424<br>A = 392+32
BDNF	11p14.1	rs6265<br>Val66Met	F: 5'-AGAAGAGGAGGCTCCAAAGG-3'<br>R: 5'-CCATTCACGCTCTCCAGAGT-3'	55°C/ 45 sec., 197 bp	*FaeI*<br>C = 197<br>T = 122+75
CIDEA	18p11.21	rs8092502<br>C>T	F: 5'-GCATCCTCCCTTGTCAAAGA-3'<br>R: 5'-TGACTGCTGGTGGTTTGGA-3'	56,3°C/ 45 sec., 490 bp	*SSe9I*<br>T = 237+163+90<br>C = 327+163
PPARGC1A	4p15.2	rs12650562<br>C>T	F: 5'-TTGAGTTCCAAAGCTGATTCC-3'<br>R: 5'-TCCAATTCCCTCAAGCTGTACGATGGCACAACG-3'	62°C/ 45 sec., 367 bp	*Bst4CI*<br>C = 334+33<br>T = 367
